# A Case of an Elderly Patient With Rubber Band Syndrome

**DOI:** 10.1016/j.jhsg.2021.07.005

**Published:** 2021-08-11

**Authors:** Erica Amemiya, Kazuhiro Maeda, Takayuki Nemoto, Iris Wiederkehr, Takeshi Miyawaki, Mitsuru Saito

**Affiliations:** ∗ Department of Orthopaedic Surgery, The Jikei University School of Medicine, Tokyo, Japan; † Hand Surgery Center, The Jikei University School of Medicine, Tokyo, Japan; ‡ Department of Orthopaedic Surgery, Ota General Hospital, Kawasaki-shi, Japan; § Department of Plastic and Reconstructive Surgery, The Jikei University School of Medicine, Tokyo, Japan

**Keywords:** Carpal tunnel syndrome, Circumferential scar, Cognitive impairment, Rubber band

## Abstract

Rubber band syndrome is a relatively rare disease in which a rubber band around a limb becomes embedded under the skin, resulting in tissue damage. Most reported cases are in children, and its occurrence in adults is considered extremely rare**.** We present a case of a 71-year-old patient with cognitive impairment, in whom a rubber band around the wrist became embedded under the skin. The examination of the distinctive circumferential scar, ultrasonography, x-ray, and magnetic resonance imaging led to the diagnosis of rubber band syndrome. To avoid further damage to the tissue, surgical removal of the band was conducted. When elderly patients with cognitive impairment present with chief complaints of swelling and contracture in the limbs due to an unknown cause, accompanied by a circumferential scar on the affected limb, rubber band syndrome should be considered. Due to risk of deep tissue necrosis, prompt band removal is necessary.

Rubber band syndrome (RBS) is a relatively rare condition in which a rubber band around a limb becomes embedded under the skin, causing tissue damage. Most reported cases are in children, and its occurrence in adults is considered extremely rare. In this report, we present the case of a patient with RBS in whom a rubber band around the wrist became embedded under the skin.

## Case Report

The patient was a 71-year-old man, who experienced swelling, tingling, numbness, and difficulty performing finger movements using his left hand. Two weeks after the onset of symptoms, the patient visited a local hospital, where he was diagnosed with cellulitis and received antimicrobial therapy. However, there was no improvement in his symptoms, and he was referred to our hospital 1 month after initial onset of symptoms.

**Figure 1:**
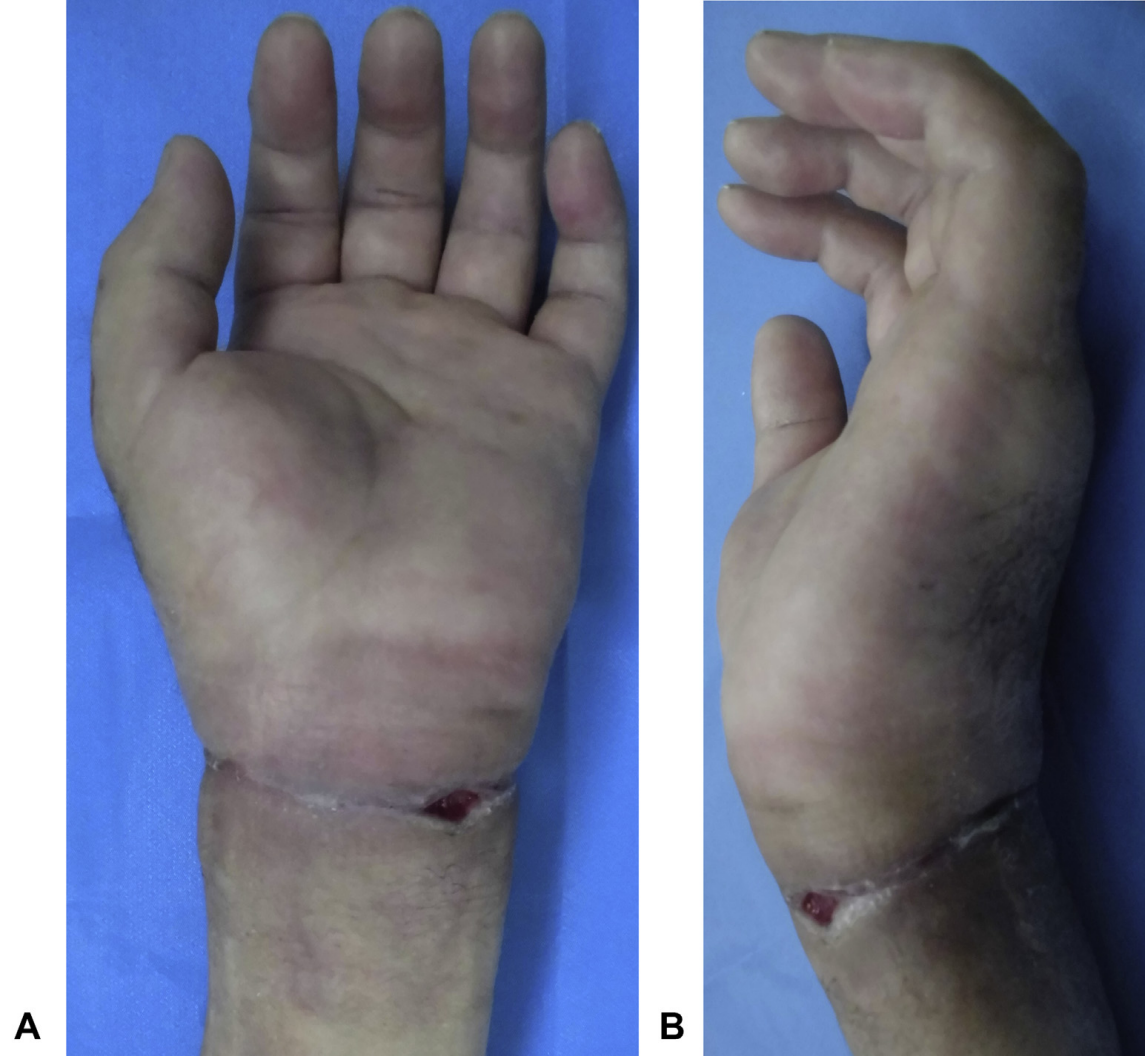
Preoperative photographs. **A** Palmar view showing a circumferential scar on the proximal wrist crease and a skin ulcer on the side of the palmar joint. **B** Lateral view showing hand contraction in an intrinsic minus position.

**Figure 2:**
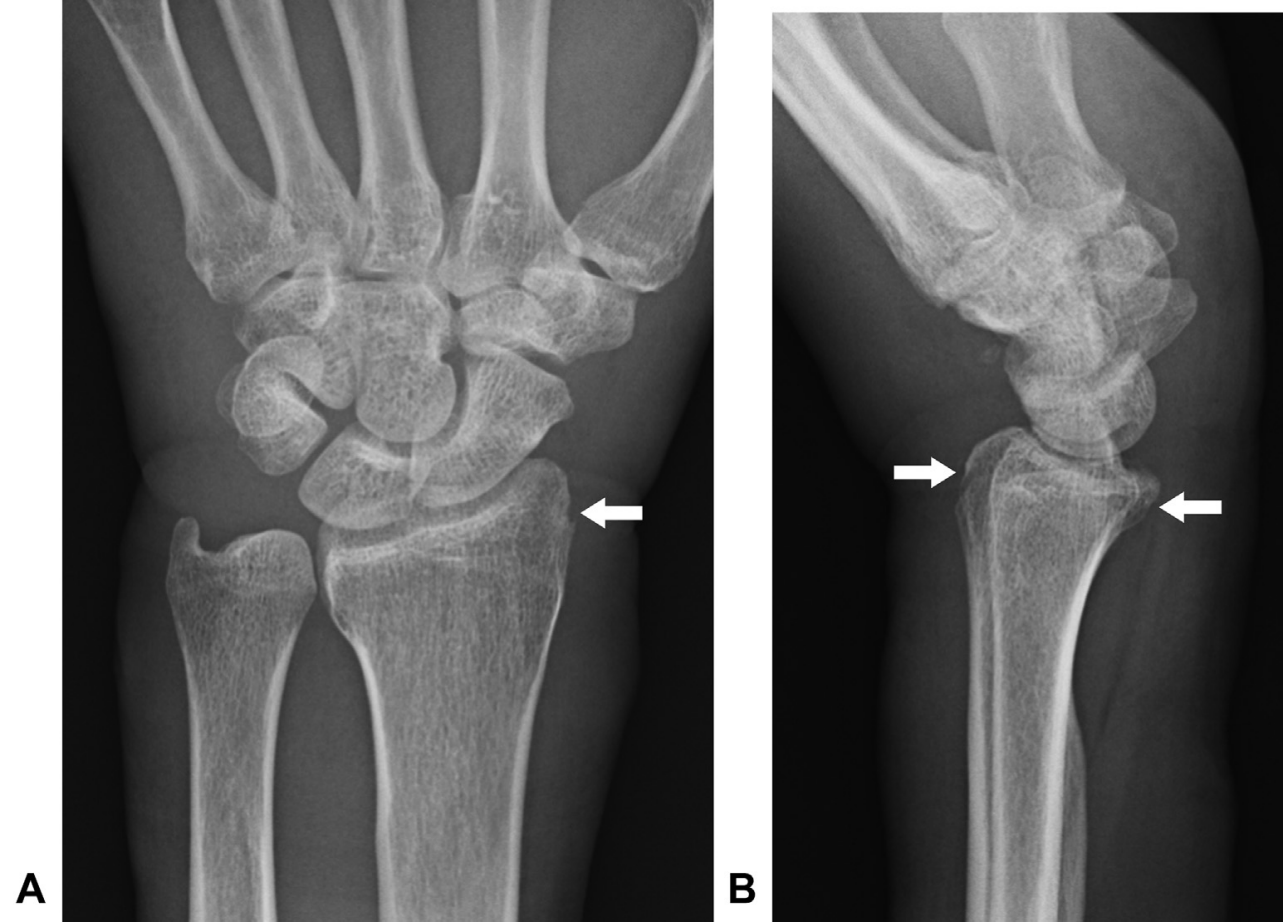
Preoperative plain radiographs confirming slight indentation on the radial bone (arrows). **A** Anteroposterior view. **B** Lateral view.

**Figure 3:**
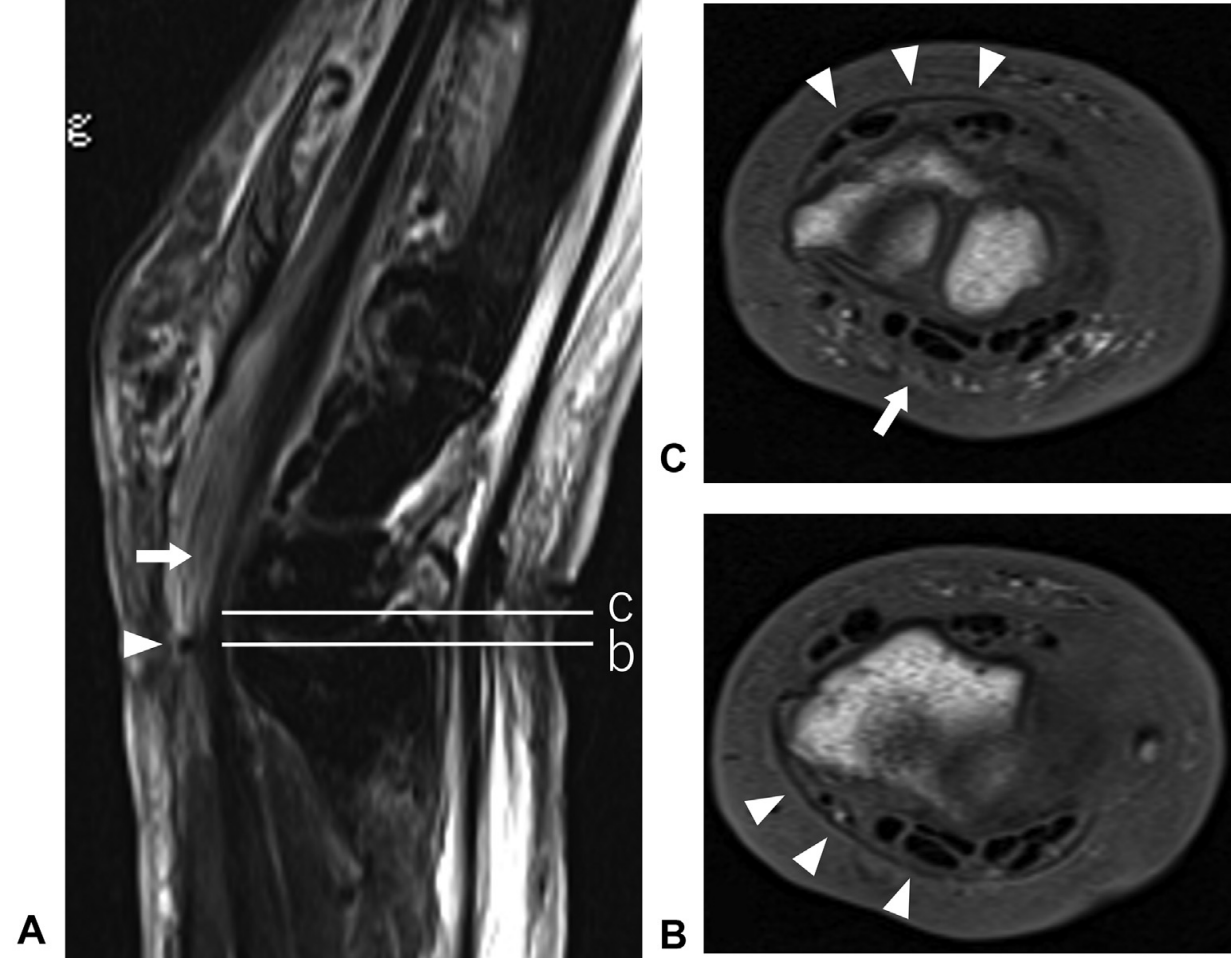
Preoperative MRI. **A** Sagittal T2-weighted imaging showing edematous changes in the whole hand and a cord-like object (arrowhead) compressing the median nerve (arrow). **B** Axial T1-weighted imaging at the level of nerve entrapment showing a cord-like object on the volar side (arrowheads); the median nerve is not visible due to entrapment. **C** Axial T1-weighted imaging at 1 slice distal from image **B**, showing a cord-like object on the dorsal side (arrowheads), with the median nerve visible on the volar side (arrow).

The initial examination showed swelling, induration, and hypesthesia of the whole left hand. The range of motion for the affected wrist was flexion of 0° (compared with healthy wrist flexion of 65°) and dorsal flexion of 60° (compared with healthy wrist dorsal flexion of 80°), indicating notable restriction in flexion. The hand was contracted in an intrinsic minus position. There was no redness or warmth. There was a circumferential scar on the proximal wrist crease and a skin ulcer on the side of the palmar joint (Fig. 1). Blood tests indicated a white blood cell count of 8,290/μL with 69% neutrophils, C-reactive protein level of 0.05 mg/dL, and blood glucose level of 102 mg/dL. There were no findings indicative of infection or diabetes. Plain radiography confirmed a slight indentation on the radial bone (Fig. 2). Magnetic resonance imaging (MRI) confirmed edematous changes in the whole hand, entrapment of the median nerve in the wrist joint, and a cord-like object compressing the wrist (Fig. 3). This led to the suspicion of constriction of the wrist joint by a cord-like object; therefore, an emergency surgery was performed.

**Figure 4:**
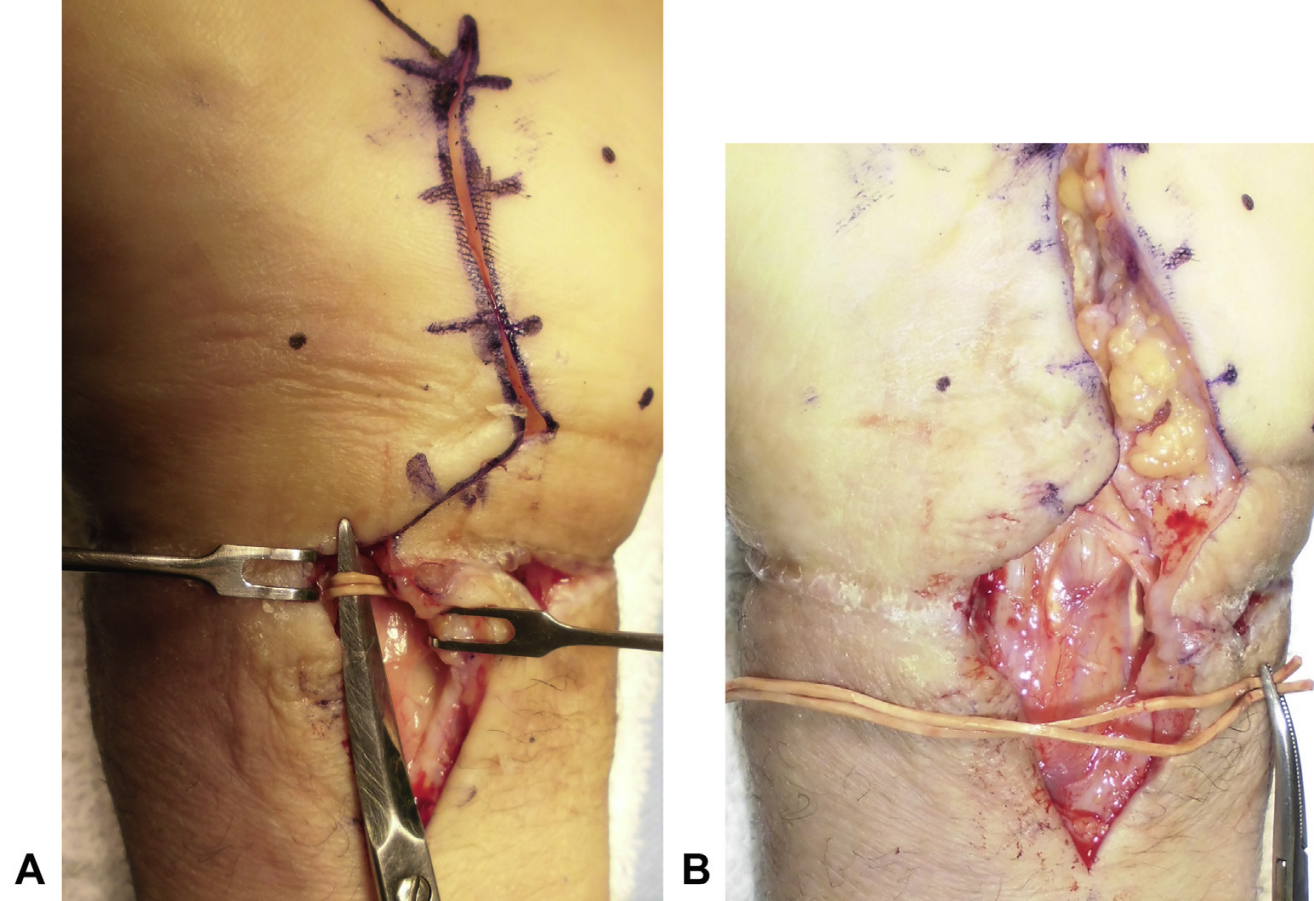
Intraoperative photographs. **A** The rubber band constricting the median nerve before removal. **B** The actual size of the rubber band after removal.

**Figure 5:**
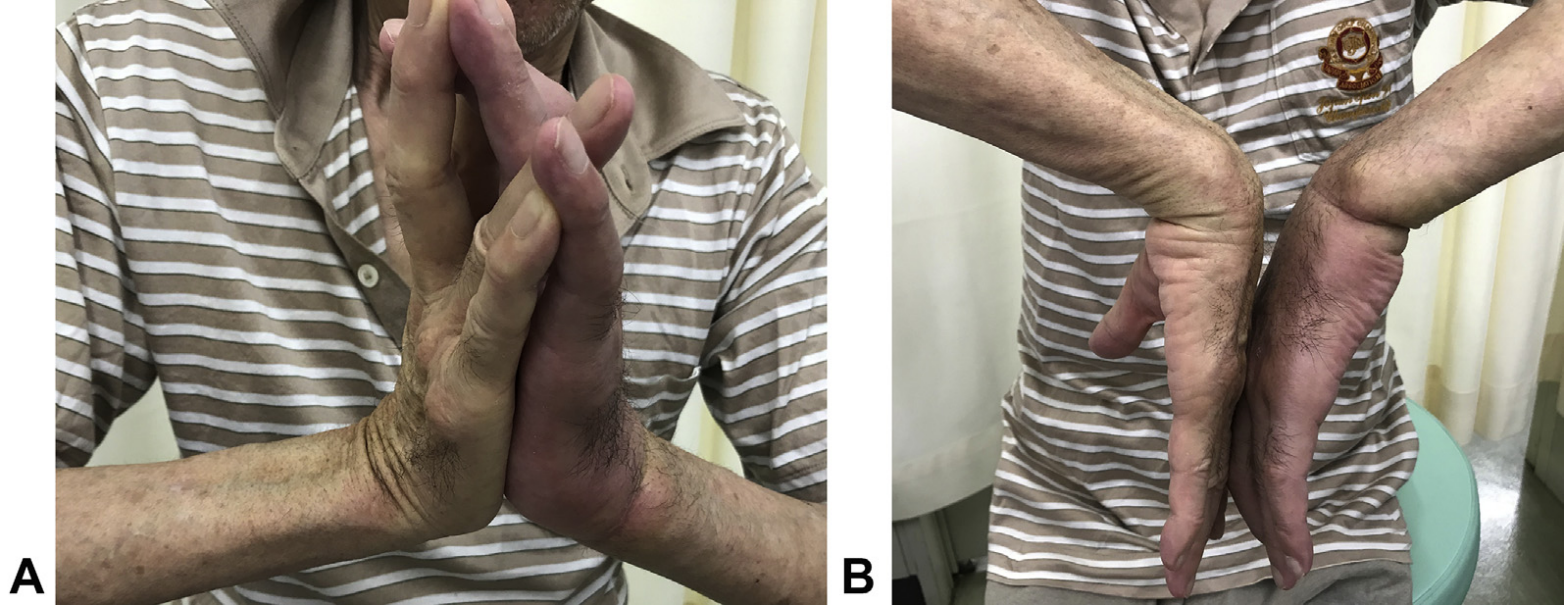
Postoperative photographs showing range of motion of the wrists at 1 year after surgery. **A** Dorsal flexion of 47°/65° (affected/healthy wrist) and **B** flexion of 66°/80°.

A zigzag incision was made on the palmar joint side, and 2 rubber bands were found embedded circumferentially under the skin, matching the scar on the proximal wrist crease. After the rubber bands were removed and the transverse carpal ligament was separated, a widespread adhesion of the flexor tendons in the carpal tunnel was discovered. The median nerve had been considerably constricted by the rubber bands (Fig. 4), and flexor tendolysis and median nerve neurolysis were performed. The patient started rehabilitation with range of motion exercises for the fingers from the first postoperative day, followed by exercises for the wrist from the second postoperative week onward. The skin ulcer epithelialized 3 weeks after surgery. Extensor tendolysis was scheduled for the third week after surgery; however, the patient expressed a strong preference for conservative therapy. Therefore, hand therapy was conducted, and to resolve the remaining intrinsic minus position of the hand, knuckle bender orthosis was applied, with which an intrinsic plus position was secured. At 1 year after surgery, the pain and swelling had subsided completely; however, a slight numbness remained in the pulp of the thumb and the index, middle, and ring fingers. The range of motion for the affected/healthy wrist was as follows: flexion of 47°/65°, dorsal flexion of 66°/80°, pronation of 80°/80°, and supination of 90°/90°. Although the patient still exhibited some movement restriction on the affected side compared with the healthy side, his condition had improved (Fig. 5). Grip strength and pinch strength improved to 9.8 kg and 3.4 kg, respectively, at 1 year after surgery compared with 4.9 kg and 2 kg immediately after surgery. In comparison, the grip strength of the healthy side was 20.7 kg and the pinch strength was 3.5 kg. Because of the emergency of the situation, no nerve conduction studies were performed before surgery. When examined 3 weeks after surgery no nerve conduction was detectable; however, 7 months after surgery nerve conduction velocity became detectable and showed a tendency to recover.

## Discussion

To date, only few studies have reported on constriction marks on the skin caused by rubber bands. Common sites for such occurrences are the neck, limbs, and the penis. There are several reported cases of infants and toddlers who accidentally wrapped a band around their neck.^1^ Cases concerning the limbs included the use of rubber bands to hold sleeves and socks in place.^2^ There are also a few case reports of rubber bands wrapped around the penis to prevent incontinence. Hogeboom and Stephens^3^ were the first to report on RBS in 1965; since then, to our knowledge, there have been 5 reported cases of adults with RBS of the upper extremity (Table 1).^1^^,^^2^^,^^4^, ^5^, ^6^ The affected sites were fingers in 4 cases and the upper arm in 1 case. Since most reported cases are in children, RBS in adults is considered to be extremely rare.^7^ Following the publication of several adult cases in the United Kingdom in the 1960s, most reports of RBS have been in children in India.^1^^,^^2^^,^^4^ In India, there is a tradition of tying a rubber band or a string around the wrist of young children for religious ceremonies and decorative purposes, which could be a causal factor.^7^ The high prevalence of RBS in children, older individuals, and people with cognitive disability could be because they do not understand the risk of having a rubber band in place, they have difficulty communicating, or they are unclear on the process of injury that could occur because of problems with memory. Since the patient in the present case underwent emergency surgical intervention, the patient’s cognitive impairment had not been assessed before surgery. After surgery, the patient appeared to have no recollection of wearing a rubber band around his wrist, which suggested a certain degree of cognitive impairment. The patient’s cognitive status was assessed using the Revised Hasegawa Dementia Scale, and he was diagnosed with mild dementia (19 points on the Revised Hasegawa Dementia Scale). Furthermore, as the patient was not able to reproduce the instructions given during hand therapy, his performance was evaluated as having “poor understanding.” In cases of severe cognitive impairment, where a caregiver is present, a rubber band may be more likely to be noticed than in people with mild cognitive impairment who are independent in daily life activities but are not well integrated into a community.

**Table:** Five Reported Cases of Adults With RBS of the Upper Extremity

Author	Age	Sex	Location	Duration	Country
Thurston^1^	Unknown (adult)	Unknown	Finger	Unknown	United Kingdom
Turney^2^	Unknown (adult)	Unknown	Upper arm	Unknown	United Kingdom
Dawson-Butterworth et al^4^	42 years	Male	Finger	3 days	United Kingdom
Whitaker et al^5^	68 years	Female	Finger	Unknown	United Kingdom
Maharjan et al^6^	Unknown (adult)	Unknown	Finger	Unknown	Nepal

An acute type of RBS can result in compartment syndrome, and in some cases, fasciotomy and carpal tunnel release has been performed subsequently.^8^ Other reports are all of the chronic type. In chronic RBS, the rubber bands cut into the soft tissue and the condition progresses gradually and is mostly painless.^7^ Rubber bands become embedded deeply under the skin through sustained tensile force, and long-term irritation leads to the development of a linear circumferential scar along the foreign object. It continues to penetrate the fascia, tendons, neurovascular structure, and osseous tissue, leading to distal edema, loss of function, infections, and neurovascular injuries.^7^ The depth of impact by rubber bands is affected by various factors such as the size of the affected site, size and strength of the rubber bands, and length of the time period between symptom onset and band removal. The longer the time period, the deeper the rubber band can reach. A circumferential scar accompanied by a fistula or ulcer is characteristic of RBS and has been mentioned in almost all reports. Imaging diagnosis usually consists of plain radiography, ultrasonography, and MRI. Because x-ray absorption by the rubber and soft tissue is similar, a definitive diagnosis is difficult on the basis of radiography imaging alone. However, in cases where osteolytic indentation or osteomyelitis is present, radiography can be used to establish the diagnosis.^9^ Ultrasonography and MRI are useful for diagnosing contracture of the soft tissue when the rubber band has penetrated the tendons and neurovascular structures.^9^^,^^10^

In all reported cases, the treatment was surgery. During incision, there is a risk of severing the rubber band; therefore, an S-shaped or a zig-zag incision is recommended instead of a vertical incision.^9^ The need for amputation of a necrotic finger has been reported previously; nevertheless, most other cases had a favorable postoperative course, and a large-scale reconstruction of tendons, nerves, and bones was not necessary.^6^ In the present case, extensor tendolysis was scheduled at 3 weeks after surgery, but the patient expressed a strong preference for conservative therapy, and the operation was not performed. Nevertheless, a good range of motion was obtained through orthosis fabrication and rehabilitation.

When elderly patients with cognitive impairment present with chief complaints of swelling and contracture in the limbs due to an unknown cause, accompanied by a circumferential scar on the affected limb, RBS should be considered. In order to establish a diagnosis, the characteristic circumferential scar, ultrasonography, and MRI are useful. This syndrome involves the risk of deep tissue necrosis; thus, an early extraction is necessary.
